# Weed Identification by Single-Stage and Two-Stage Neural Networks: A Study on the Impact of Image Resizers and Weights Optimization Algorithms

**DOI:** 10.3389/fpls.2022.850666

**Published:** 2022-04-25

**Authors:** Muhammad Hammad Saleem, Kesini Krishnan Velayudhan, Johan Potgieter, Khalid Mahmood Arif

**Affiliations:** ^1^Department of Mechanical and Electrical Engineering, School of Food and Advanced Technology, Massey University, Auckland, New Zealand; ^2^Massey AgriFood Digital Lab, Massey University, Palmerston North, New Zealand

**Keywords:** deep learning, convolutional neural network, weed detection, optimization algorithms, transfer learning

## Abstract

The accurate identification of weeds is an essential step for a site-specific weed management system. In recent years, deep learning (DL) has got rapid advancements to perform complex agricultural tasks. The previous studies emphasized the evaluation of advanced training techniques or modifying the well-known DL models to improve the overall accuracy. In contrast, this research attempted to improve the mean average precision (mAP) for the detection and classification of eight classes of weeds by proposing a novel DL-based methodology. First, a comprehensive analysis of single-stage and two-stage neural networks including Single-shot MultiBox Detector (SSD), You look only Once (YOLO-v4), EfficientDet, CenterNet, RetinaNet, Faster Region-based Convolutional Neural Network (RCNN), and Region-based Fully Convolutional Network (RFCN), has been performed. Next, the effects of image resizing techniques along with four image interpolation methods have been studied. It led to the final stage of the research through optimization of the weights of the best-acquired model by initialization techniques, batch normalization, and DL optimization algorithms. The effectiveness of the proposed work is proven due to a high mAP of 93.44% and validated by the stratified k-fold cross-validation technique. It was 5.8% improved as compared to the results obtained by the default settings of the best-suited DL architecture (Faster RCNN ResNet-101). The presented pipeline would be a baseline study for the research community to explore several tasks such as real-time detection and reducing the computation/training time. All the relevant data including the annotated dataset, configuration files, and inference graph of the final model are provided with this article. Furthermore, the selection of the DeepWeeds dataset shows the robustness/practicality of the study because it contains images collected in a real/complex agricultural environment. Therefore, this research would be a considerable step toward an efficient and automatic weed control system.

## Introduction

With the fast-growing global population, food demand is expected to increase up to 70% by 2050 (Caldera and Breyer, 2019). Therefore, various challenges in the agricultural sector have been addressed by the research community to get smart and intelligent solutions. Among various agricultural problems, weeds are a serious threat to crop yield that causes economic loss (Ahmad et al., 2021). An effective way to manage the weed is to use herbicide spray specifically in the field that contains the weeds. Accurate and precise detection of weeds is important to successfully deploy the weed management system (Hasan et al., 2021). This agricultural task is time-consuming and requires a great amount of human and machine resources. Furthermore, fast and automatic identification of weeds is essential to reduce excessive/unrequired application of a chemical spray that produces adverse effects on human beings and ecosystems (Lottes et al., 2020).

After the introduction of the AlexNet model in 2012 (Krizhevsky et al., 2012), deep learning (DL) has recognized its ability to detect, classify, and localize several objects quickly. The object identification tasks are performed in controlled/laboratory and uncontrolled/real environments. Similarly, the agricultural field of research is being accelerated by leveraging various developments in DL. The research community is extensively focusing on agricultural tasks including fruit harvesting/recognition (Fu et al., 2021; Gai et al., 2021), plant recognition (Quiroz and Alférez, 2020; Bisen, 2021), identification of crop water stress (Chandel et al., 2021), land cover classification (Saleem et al., 2021), and plant disease detection (Priyadharshini et al., 2019; Saleem et al., 2019; Uguz and Uysal, 2021) by investigating DL-based techniques. Similarly, recent advances in DL have encouraged researchers to address the classification and detection of weeds in several plant species (Hasan et al., 2021).

On the other hand, after rapid developments in DL, still, the robustness of the DL-based solutions is a critical research question among the scientific community. There are various aspects to realize the strength of DL, and the environment of the collected dataset images is one of the important factors. It is commonly observed that DL architectures provide higher accuracy on the images collected in controlled or with plain/single background compared to those which were collected in a real environment. This is due to the presence of various unessential or background elements/objects that could have characteristics similar to the required objects. Furthermore, occlusion is another aspect of reducing or degrading the performance of the DL models. Therefore, agricultural researchers and data scientists started collecting images for the datasets in real agricultural environments. To address the concerns described above, a publicly available dataset called DeepWeeds (Olsen et al., 2019) has been used throughout this research, which contains various characteristics of the real agricultural environment.

A summary of prominent studies regarding the identification of weeds by various methodologies related to DL is presented in Table 1. It can be concluded that previous studies focused on DL-based weed detection in four ways: evaluation of transfer learning techniques, investigation of the performance of state-of-the-art DL models, integration of DL models with other image processing-based/traditional machine learning methods, and modification of the well-known DL architectures. To the best of the authors' knowledge, none of the previous articles has provided a comprehensive study of weed detection by performing an in-depth analysis of single- and/or two-stage DL-based object detectors along with an extensive investigation of various aspects of DL in terms of image resizers, image interpolation, weight initialization, batch normalization, and optimization methods. The major contributions of this study are: (1) a novel DL-based methodology is presented to identify the weeds by analyzing and evaluating the performance of various single-stage and two-stage neural networks; (2) also, the effects of various image resizing techniques are discussed. Moreover, weights of the best-obtained neural network are optimized with initialization method, batch normalization, and optimization algorithms; their effects on the training and testing datasets are also thoroughly studied; (3) the optimized/modified DL approach improved the mean average precision (mAP) with a significant margin as compared to the default settings; attained enhanced average precision in individual classes; the presented approach can be adapted to other agricultural operations due to a high mAP for weed detection; (4) an in-depth analysis of the best-obtained DL architecture is performed; it has provided a strong basis of the future research to propose a modified DL model for further enhancing the research on weed detection; (5) the trained weights of various DL models can also be used as transfer learning for other weed-related datasets. Moreover, the proposed methodology can be treated as an earlier step before modifying the hidden layers of neural networks for other agricultural applications.

**Table 1:** Summary of research articles related to weed detection by deep learning (DL) (divided in terms of novelty and research ideas of the work).

**Novelty/research ideas**	**DL models**	**Number of classes**	**Dataset conditions**	**Best model performance**	**References**
Investigation of DL models for the identification of weeds	AlexNet, GoogLeNet, VGGNet, DetectNet	Three	Various surface condition regimes.	DetectNet F1-score = 0.9843	Yu et al., 2019a
DL architectures were leveraged for weed detection and classification	DetectNet, GoogLeNet, and VGGNet	Three	Different stages and densities of growth	F1-score by DetectNet > 0.99	Yu et al., 2019b,c
Speed-optimized CNN models were proposed	CNN model	Two	Images were taken with a field robot in a real environment.	A speed-up factor of 31	Knoll et al., 2019
DL model used with color index-based segmentation	CenterNet	One	Different illumination conditions, backgrounds, and growth stages	F1-score by the CenterNet model = 0.953	Jin et al., 2021
A tiny version of the YOLO model was proposed to reduce the computation time	Modified tiny YOLO-v3, YOLO-v3-tiny	Two	Synthetic images were generated	Mean average precision: 0.829	Gao et al., 2020
Various factors to develop weed identification system along with the significance of transfer learning	AlexNet, VGG-F, VGG-VD-16, Inception-v1, ResNet-50, ResNet-101	Two	A robotic platform was used to take images on the field.	Accuracy by ResNet-101: 97.1+/-0.1%	Kounalakis et al., 2019
Two DL detectors were used through a UAV	Faster RCNN and SSD	Six	Images were taken by a camera mounted on a UAV	Mean IoU by Faster RCNN: 0.85	Veeranampalayam Sivakumar et al., 2020
An improved DL model was proposed	Proposed Faster RCNN, KNN, SVM, and YOLO-v3	Two	A camera mounted on a UAV in two agricultural fields	Overall average identification accuracy: 94.7%	Khan et al., 2021
A CNN model was optimized for real-time weed recognition	ResNet-18	Six	Dataset images were collected by a UAV	Overall accuracy: 94%	De Camargo et al., 2021
Three ML and DL-based methods were used and compared	SVM, YOLO-v3, and Mask R-CNN	Two	A multispectral camera mounted on a drone was used	F1-score by YOLO and RCNN models: 94%	Osorio et al., 2020
DL-based classification and detection models were used	VGG-16, ResNet-50, Inception-v3, YOLO-v3	Four	Images were collected in a real field environment	mAP: 54.3%	Ahmad et al., 2021
A graph CNN-based model was proposed to detect weeds	GCN-ResNet-101, AlexNet, ResNet-101, VGG-16	Four	The Weeds were collected in three crops and a fourth was obtained by combining the three datasets.	Average recognition accuracy: 98.15%	Jiang et al., 2020
A combination of DL and ML methods was considered	Xception, Inception-ResNet, VGNets, MobileNet, DenseNet, SVM, XGBoost, and Logistic Regression	Two	The dataset was collected under variable soil, color and illumination conditions.	F1-score: 99.29%	Espejo-Garcia et al., 2020

## Materials and Methods

**Figure 1:**
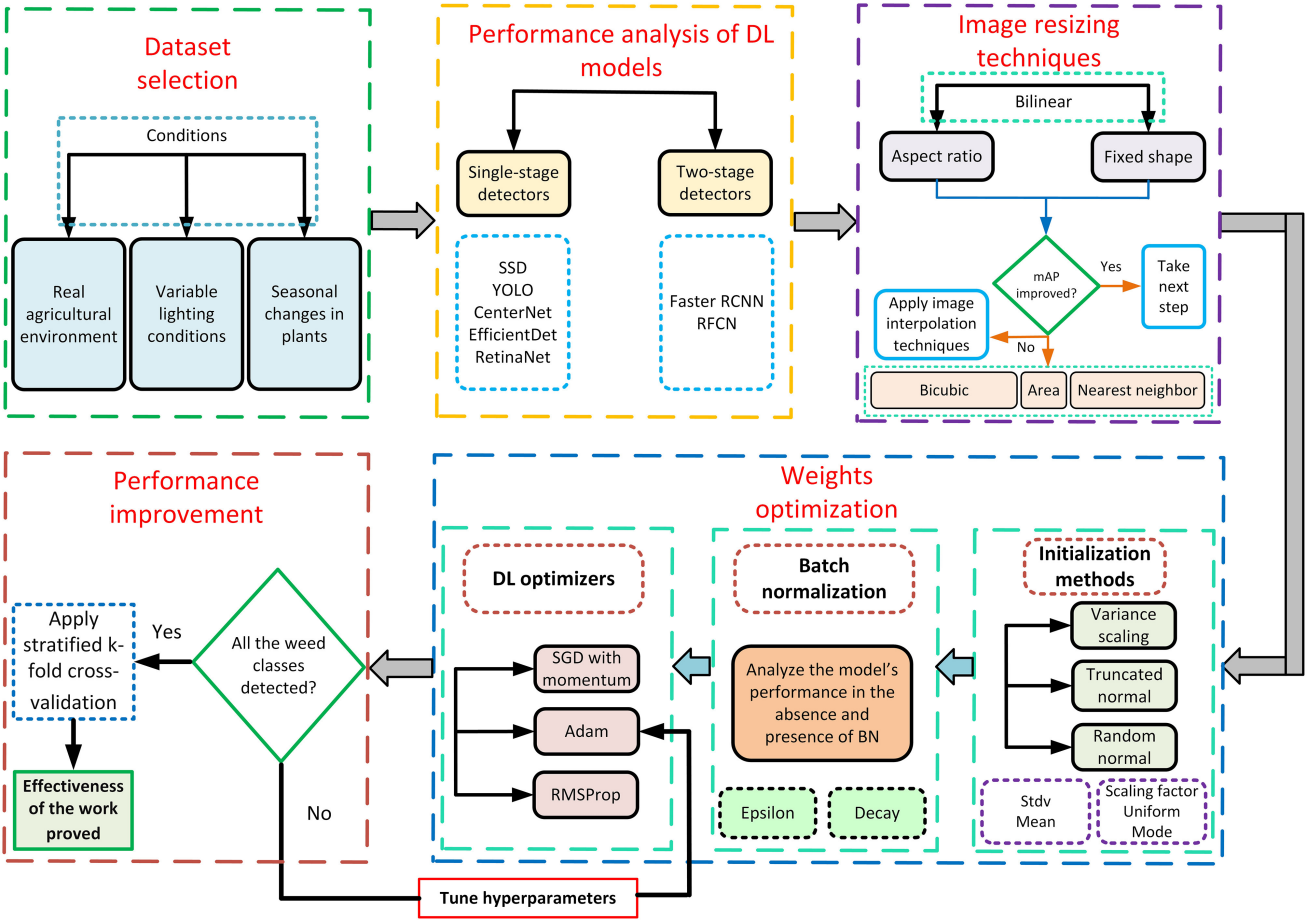
Framework of this research.

This article presents a DL-based approach to detect and classify eight types of weeds. First, a publicly available dataset called the DeepWeeds dataset is selected, which covers different aspects of a real agricultural environment. The proposed method comprises four steps. The first step is the analysis of various single-stage and two-stage object detectors and the best-suited DL model which attained the highest mAP. It led to the empirical evaluation of image resizing techniques like aspect ratio and fixed shape resizers by using the image interpolation methods including bilinear, bicubic, area, and nearest neighbor. Then, an attempt was made to optimize the weights of the DL model in two stages. First, the parameters of the weight initialization methods, such as the truncated normal, variance scaling, and random normal techniques, were studied. Later, the effects of batch normalization were studied. Finally, adaptive DL optimization techniques including Adam and RMSProp were applied to further enhance the performance of the best-obtained DL architecture as presented for other agricultural applications (Saleem et al., 2020a,b); their hyperparameters were tuned with the random search method. The final mAP was compared with the one obtained by default settings to show the effectiveness of this research. The obtained mAP was validated by the stratified k-fold cross-validation method. An overall methodology is also presented in Figure 1.

### Selection of the Dataset

The main criteria for selecting the dataset were the images should be collected in a real agricultural environment considering various features of the actual field. These characteristics include natural background, occlusion, rotation, geographical/seasonal changes in plants, and variable lighting conditions. As the DeepWeeds dataset (Olsen et al., 2019) had all these characteristics, it was selected for this research. These conditions were important to consider because a higher accuracy attained on this kind of dynamic dataset would prove the robustness of the proposed work. The dataset contains 17,509 images divided into eight classes of weeds, including one negative class that has non focused plants, and the images were collected in Northern Australia.

### Dataset Division and Annotation

**Figure 2:**
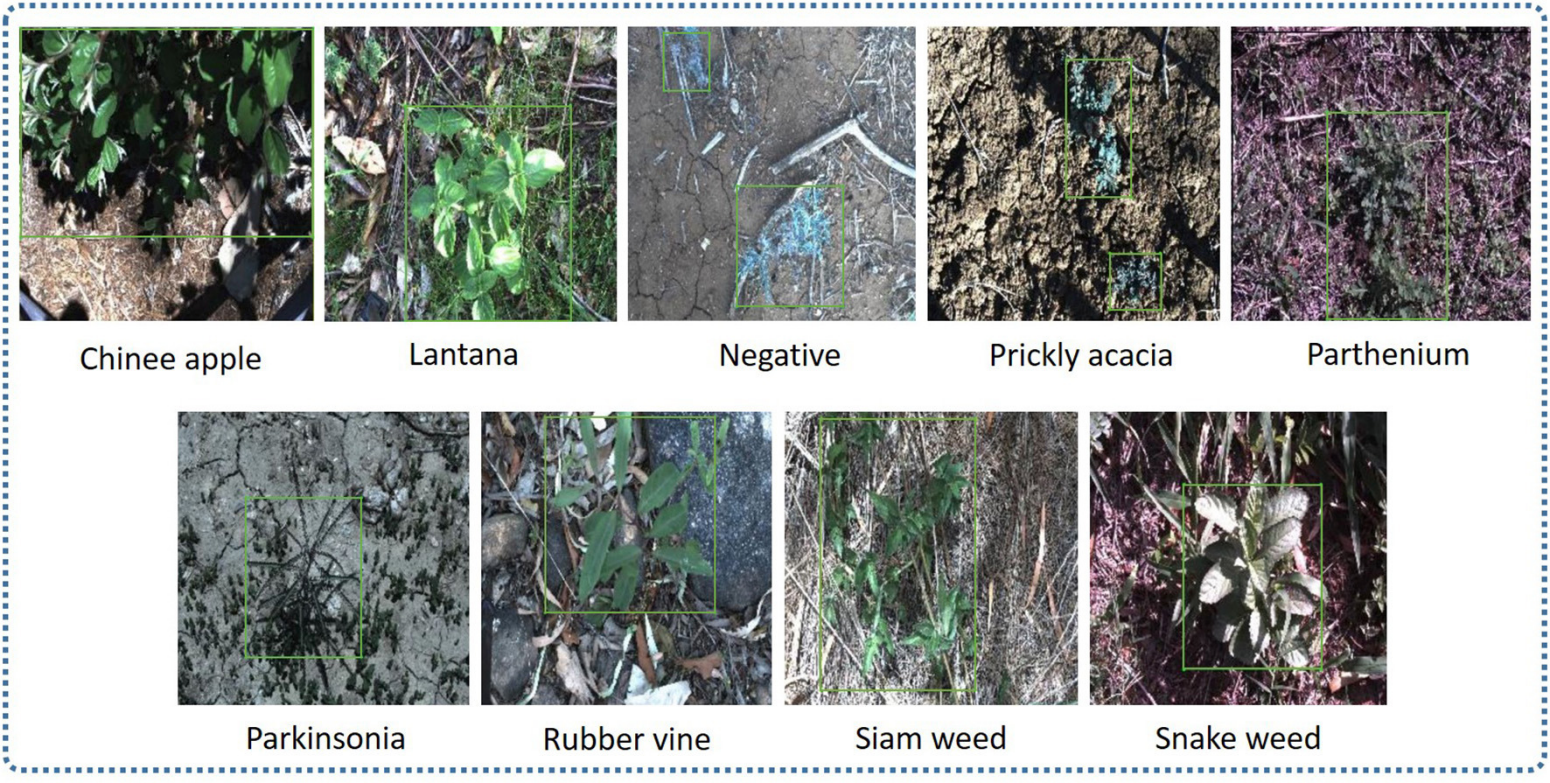
Sample of the annotated dataset for each class.

The DeepWeeds dataset was divided into three sub-datasets: training (70%), validation (20%), and testing (10%). For models like Single-shot Multibox Detector (SSD), RetinaNet, Faster Region-based Convolutional Neural Network (RCNN), Region-based Fully Convolutional Networks (RFCN), CenterNet, and EfficientDet in TensorFlow object detection Application Programming Interface (API), the dataset images were annotated using an open-source image annotation tool called LabelImg. The bounding box coordinates were obtained in Xmin, Xmax, Ymin, and Ymax. The annotations were saved in XML format, which was converted to CSV format and later to TF records (Saleem et al., 2020a). Unlike TensorFlow models, images annotated in XML format were then converted to TXT format to train the YOLO-v4 model. To visualize the detected results within a bounding box, a shortened name of each class was set to label the images before training. For example, chinee apple was replaced by C_App, lantana was replaced by Lntna, prickly acacia was replaced by P_acacia, parthenium with P_nium, parkinsonia with P_sonia, rubber vine with R_vine, siam weed with S_weed, snakeweed with Snk_wd, and negative with Ngtv. A sample of annotated images of each class is presented in Figure 2.

### DL-Based Object Detectors

In this article, the performance of various versions of DL models integrated with different feature extractors or backbone models were analyzed on the selected dataset. These DL meta-architectures are divided into two categories: single-stage and two-stage neural networks. Among single-stage detectors, state-of-the-art models like SSD (Liu et al., 2015), CenterNet (Duan et al., 2019), EfficientDet (Tan et al., 2019), RetinaNet (Lin et al., 2017), and YOLO-v4 (Bochkovskiy et al., 2020) were included. These models were trained using TensorFlow (1 and 2) object detection API and the YOLO/Darknet neural network framework. The two-stage DL models were also trained and tested using TensorFlow Object Detection API 1. These networks generally contain the first stage of the region proposal by Region Proposal Network (RPN). While the second stage refines the classification and localization of the proposals. The most prominent among them were the Faster RCNN (Ren et al., 2015) and RFCN (Dai et al., 2016) models.

### Image Resizing Techniques

The second step of the proposed method was the evaluation of the best-obtained DL object detector using image resizing techniques along with image interpolation methods. In this regard, the input images were resized to a fixed shape or by using an aspect ratio having minimum and maximum image dimensions in pixels. For example, Faster RCNN used a shorter pixel value of 600 and a longer one equal to 1,000 pixels as default values. Furthermore, these image resizers were used with interpolation techniques including bilinear (bilinear interpolation), bicubic (cubic interpolation), area, and nearest neighbor (multivariate interpolation for multiple dimensions).

### Weight Initializers

The approach proposed in this article extensively considers weight optimization in three ways. First, the effects of initialization methods were analyzed depending on the type of neural network layer/activation function. Three initialization methods include truncated normal, variance scaling, and random normal initializers. The truncated normal creates a tensor having a truncated normal distribution, which is useful to avoid dead neurons due to ReLU activation functions. It discards and redraws values more than two SD from the mean. It is the most recommended weight initialization technique for neural network-based DL models.

When the ReLU activation function came after the Sigmoid function, it was proven to successfully solve the problem of vanishing gradients. Then a weight initialization technique was proposed which balances the variance of the output layer with the input layer (He et al., 2015) and is known as He initialization. In the TensorFlow Object Detection API, the He initialization is named as the variance scaling initializer. The last initializer is a random normal initializer that is used to generate tensors with a normal distribution.

### Batch Normalization

Batch normalization was introduced to solve the problem of internal covariate shift due to the change in the distribution of the input of the neural network layer with the change in the parameters of the previous layers (Ioffe and Szegedy, 2015). The use of BN increases the training speed to get the convergence of the model with a high learning rate.

### Deep Learning Optimization Techniques

The next step of the research was weight optimization using DL optimizers. Their hyperparameters were tuned with the help of the random search method. Three optimizers were used for this purpose, namely, Stochastic gradient descent (SGD) with momentum, Root mean square propagation (RMSProp), and Adaptive moment estimation (Adam). SGD (with momentum) is one of the most commonly used DL optimizers to train DL architectures for various applications (Saleem et al., 2020a) due to its fast convergence, which is a result of the inclusion of an exponentially weighted average of weights and bias gradients (Ruder, 2016). On the other hand, RMSProp limits the oscillations generated during the training by considering the square of gradients of weights and biases. Furthermore, it allows the algorithm to consider a larger learning rate (Hinton et al., 2012). Adam optimizer is the combination of RMSProp and momentum optimizers. It includes an exponentially decaying average of the previous gradient with squared gradients (Kingma and Ba, 2014).

### Stratified k-Fold Cross-Validation Technique

The DeepWeeds dataset has the class imbalance problem, for example, the negative/non-weed class has a significantly higher number of images as compared to all eight classes of weed. Therefore, a stratified k-fold cross-validation technique was selected to validate the final results. This method avoids biasness while creating the folds of training/testing dataset and allows to maintain the same proportion of each class sample in each fold, as in the initial distribution (He and Ma, 2013). It was made sure that the testing images in each fold were not the same.

### Software and Training Specifications

The DL meta-architectures were trained and tested using TensorFlow object detection API 1, 2, and YOLO/Darknet neural network framework. All experiments were carried out using a Graphical Processing Unit (NVIDIA GeForce GTX 1080 Ti) with specifications: 11 GB memory, 1,582 MHz boost clock, 3,584 CUDA cores, and 484 GB/sec memory bandwidth. CuDNN library was imported to increase the training speed.

To leverage transfer learning, the pre-trained weights on the COCO dataset were used. Depending on the DL architecture and GPU limitations, batch sizes equal to 2, 4, 6, and 8 were the most reasonable values to minimize the trade-off between mAP and training time (Masters and Luschi, 2018). The learning rate and the values of other hyperparameters were selected by the random search technique (Bergstra and Bengio, 2012) as presented in Table 2.

**Table 2:** Hyperparameters of deep learning optimization algorithms with their respective DL architectures.

**DL models**	**DL optimizers**	**Hyperparameters**
Yolo-v4	SGD with momentum	learning rate = 1 x 10^−3^, momentum = 0.9
RetinaNet		learning rate = 3 x 10^−4^, momentum = 0.9
EfficientDet		learning rate = 2 x 10^−4^, momentum = 0.9
RFCN ResNet-101		learning rate = 4 x 10^−4^, momentum = 0.9
Faster RCNN Inception-v2		learning rate = 2 x 10^−4^, momentum = 0.9
Faster RCNN ResNet-50		learning rate = 3 x 10^−4^, momentum = 0.9
SSD MobileNet	RMSProp	learning rate = 2 x 10^−3^, rho = 0.9, momentum = 0.9, epsilon = 1.0 x 10^−2^
SSD Inception-v2		learning rate = 2 x 10^−4^, rho = 0.9, momentum = 0.9, epsilon = 1.0 x 10^−4^
CenterNet ResNet-50	Adam	learning rate = 1 x 10^−3^, epsilon = 1 x 10^−7^
Faster RCNN ResNet-101	SGD with momentum	learning rate = 3 x 10^−4^, momentum = 0.9
	Adam	learning rate = 1 x 10^−5^, epsilon = 1 x 10^−2^
	RMSProp	learning rate = 3 x 10^−4^, rho = 0.9, momentum = 0.9, epsilon = 1.0

## Results and Discussion

This article aims to identify and localize eight classes of weeds using DL architectures. In this regard, seven DL architectures were trained with different feature extractors/backbone models. The performance of these architectures is evaluated in terms of mAP, which is a commonly used performance metric for object detection tasks. Equation (1) presents the formula to evaluate mAP.


(1) $$\begin{array}{r} \text{mAP}\;\text{=}\;\frac{\overset{\text{n}}{\underset{\text{i}\;\text{=}\;\text{1}}{\sum }}{\text{AP}}_{\text{i}}}{\text{n}} \end{array}$$

where AP is the average precision calculated for each class and accessed by the 11-point interpolated AP method and *n* is the number of classes. The AP is defined as the average precision across all unique recall levels. Therefore, the precision at various recall values is first evaluated. Then, interpolated precision is calculated as the maximum precision for a certain recall level. Further details of this performance metric can be found in (Saleem et al., 2020a).

This section is divided into two steps. First, the weed detection results obtained by the single- and two-stage DL architectures are provided along with their class-wise performance analysis and loss plots. Secondly, the effects of various image resizing techniques on the best-obtained DL model are presented. Furthermore, the weights were optimized by leveraging initialization techniques along with batch normalization and DL optimizers. Finally, a significant improvement in the mAP of the optimized DL architecture is discussed compared to its default settings to demonstrate the effectiveness of the proposed approach.

### Step 1: Selection of the Best-Suited DL Architecture

#### Performance of Single-Stage Neural Networks

##### YOLO-v4

**Figure 3:**
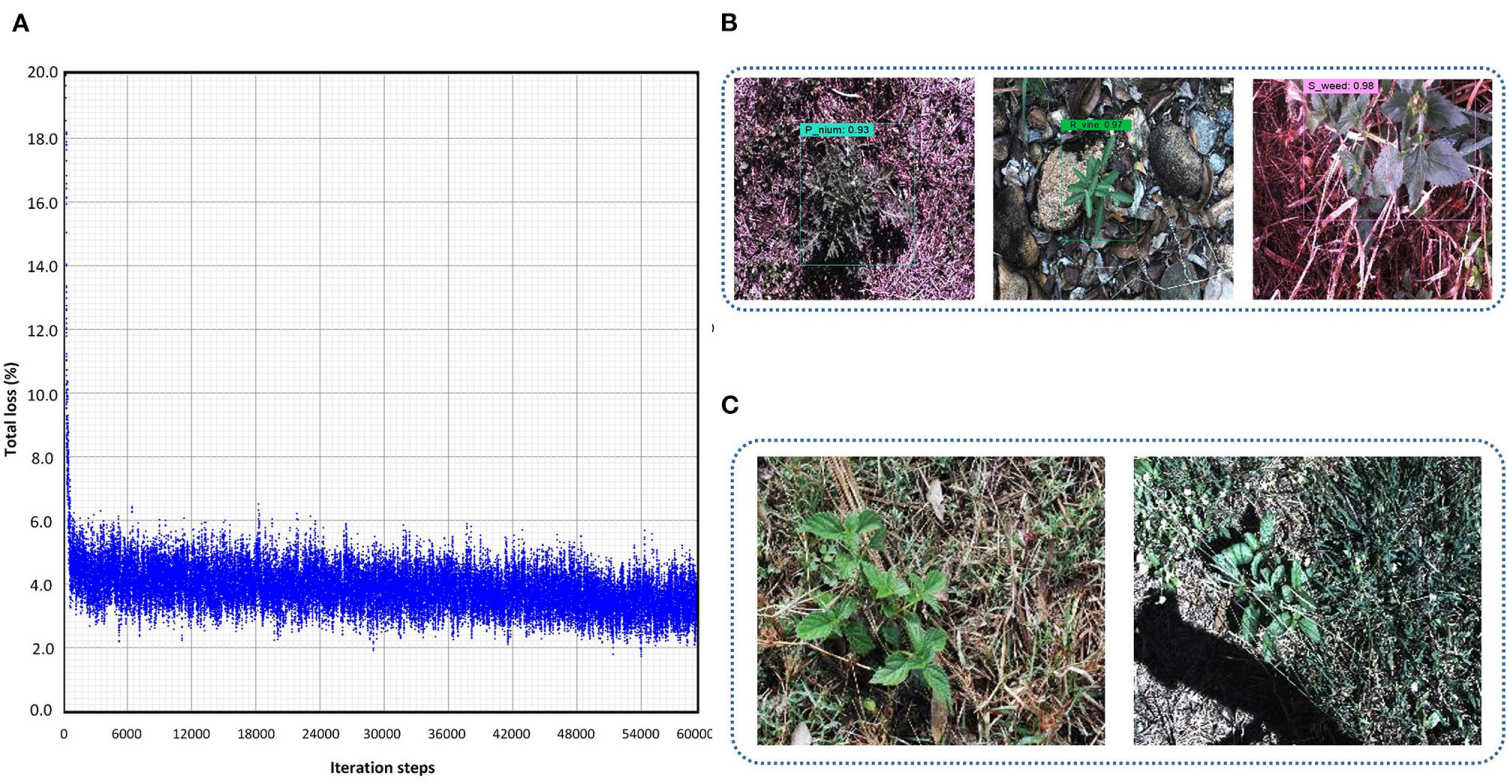
Performance of the You look only Once (YOLO)-v4 model: **(A)** loss plot; **(B)** true positives for parthenium, rubber vine and siam weed; **(C)** examples of undetected images for lantana and snake weed classes.

This DL architecture has the backbone model CSPDarknet-53 having input image size 608 x 608. The SGD with momentum optimizer was used to train the model. Various batch sizes were tested and eight was found to be the most feasible to reduce the trade-off between accuracy and training time as minimum as possible. The plot in Figure 3A shows that the model started to converge after 48K iterations, and the model training took around 12 h. The final average loss value was found to be 2.83%. A few classes of weeds were successfully identified, including parthenium, rubber vine, and siam weed, with an average precision of more than 90%. Therefore, their distinct characteristics were successfully extracted. None of the classes attained <50% AP which shows the significance of this model to detect several classes of weed. An example of the three classes which achieved the highest AP is presented in Figure 3B. Furthermore, some of the images of classes such as lantana and snakeweed were undetected, as shown in Figure 3C. The mAP of all classes was calculated to be 79.68% as shown in Table 3. Each prediction box is related to the class label with a confidence score of 0 to 100% (0 to 1).

**Table 3:** Summary of the weed detection results of the DL single-stage and two-stage object detectors in terms of the average precision (in %) of each class.

**Annotated weed and negative classes**	**DL architectures with backbone models**									
	**Single-stage networks**						**Two-stage networks**			
	**Yolo-v4 CSPDarknet-53**	**SSD**			**EfficientDet** **EfficientNet**	**CenterNet** **ResNet-50**	**RFCN** **ResNet-101**	**Faster RCNN**		
		**Inception-v2**	**MobileNet**	**ResNet-50 (RetinaNet)**				**Inception-v2**	**ResNet-50**	**ResNet-101**
C_App	67.4	26.25	18.83	45.31	43.97	26.34	34.29	100	98.21	99.87
Lntna	66.61	62.22	31.65	9.09	28.79	9.09	100	96.83	99.45	82.46
P_acacia	73.87	34.45	0.75	9.09	9.67	1.82	56.5	28.64	94.08	70.06
P_nium	93.48	54.16	26.36	17.88	33.84	23.85	38.83	99.94	99.94	99.33
P_sonia	79.51	53.93	30.92	17.05	44.23	35.77	99.7	99.24	99.89	88.85
R_vine	96.33	60.44	76.99	27.27	44.18	35.88	92.9	99.77	100	99.84
S_weed	98.6	66.06	26.35	54.55	63.29	53.31	41.61	82.49	100	99.85
Snk_wd	58.19	62.4	21.36	14.91	34.79	33.57	0.55	4.17	15.38	86.17
Ngtv	83.17	13.2	81.72	0.11	26.57	26.61	31.18	51.28	78.13	62.35
mAP (%)	79.68	48.12	34.99	21.69	36.59	27.36	55.06	73.59	87.23	**87.64**

*Bold values shows the highest mAP to select the best DL architecture*.

##### SSD

**Figure 4:**
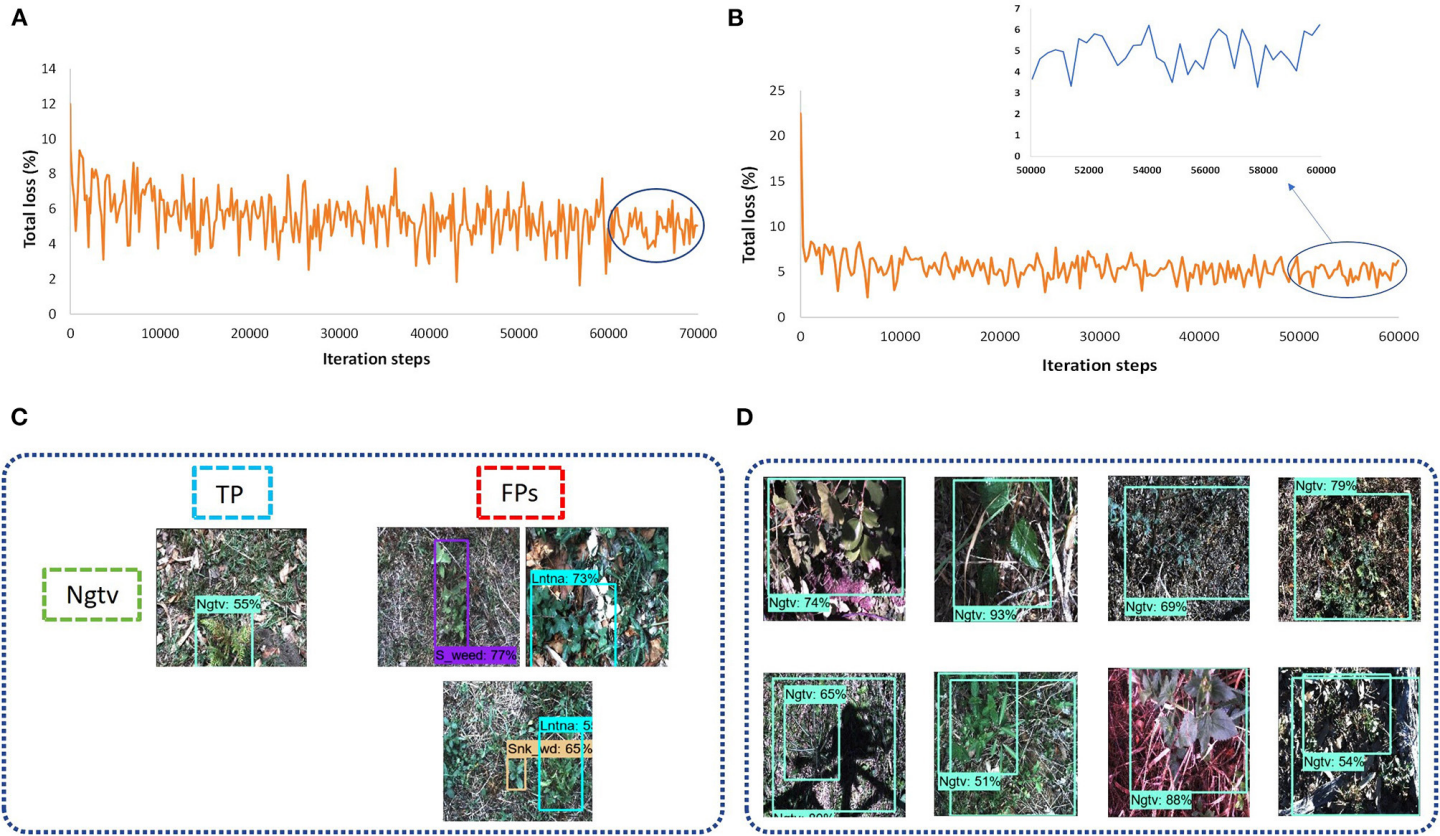
Performance of the single-shot multibox detector (SSD) architecture: **(A)** total loss with the Inception model; **(B)** total loss with the MobileNet model; **(C)** examples of a false-positive result for the negative class with the Inception-v2 model; **(D)** example of false positives for the eight classes of weeds with the MobileNet model. TP: true positive, FP: false positive.

A Single-shot MultiBox detector was trained with different backbone models to extract the features of weed classes. The feature extractors like Inception v2 and MobileNet from TensorFlow 1 API were considered. The fixed input image resizer was applied with 300 × 300 dimensions. The model was trained with an RMSProp optimizer, as the momentum optimizer was unable to converge the training. It took around 11 h to complete the training in 70K steps for the Inception model, and the batch size was equal to 8. However, the MobileNet model took around 5 h to converge the training in 60K steps, which was the fastest among all other models due to its fewer parameters (Huang et al., 2016). The total loss was fluctuated between 4 to 6% in the case of the Inception-v2 model (Figure 4A) while it was 3 to 6% for the MobileNet model (Figure 4B). From the results, it can be concluded that none of the weed classes was able to achieve an AP of more than 90%, with the SSD model trained with Inception and MobileNet feature extractors. However, the siam weed class achieved the highest AP of 66.06% with the Inception model, and the negative class achieved the lowest AP of 13.2%. The reason for the false detection of the negative class was confusion with three classes, including snakeweed, siam weed, and lantana, as presented in Figure 4C. The negative class worked well with the MobileNet model, while the prickly acacia was almost undetected, as its AP was only 0.75%. All weed classes were confused with the negative class when the feature extractor was MobileNet as shown in Figure 4D. It resulted in a lower mAP of approximately 35%, as shown in Table 3.

##### RetinaNet

**Figure 5:**
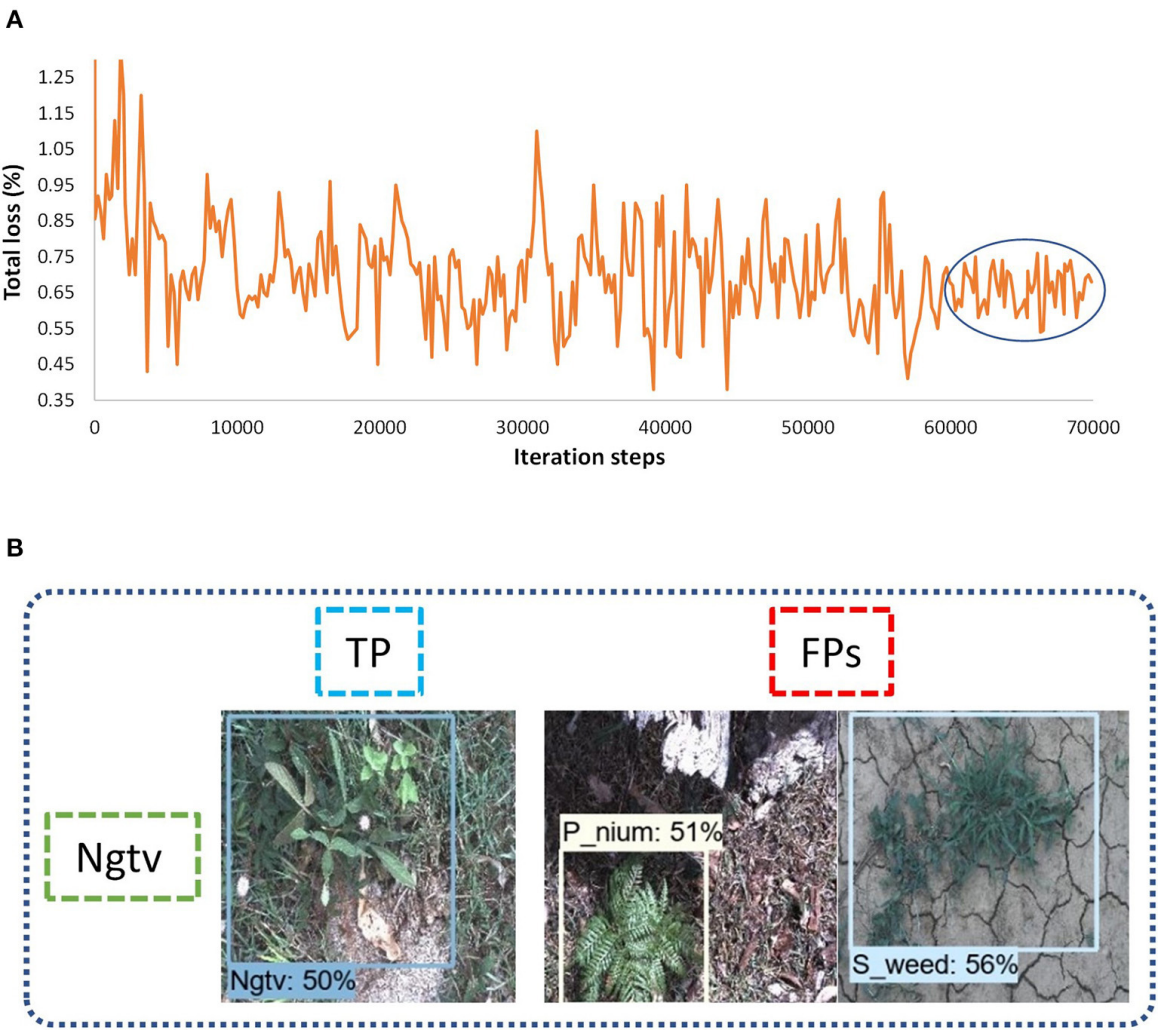
Performance of RetinaNet: **(A)** total loss plot; **(B)** examples of false-positive results for the negative class.

In this research, three DL object detectors from TensorFlow object detection API 2 were also tested. The first model was the RetinaNet, which had an SSD model as a base architecture, and ResNet-50 was used as a feature extractor. Although other versions of ResNet (with 101 and 152 layers) were also available in the API, due to GPU memory limitations, only ResNet-50 was feasible to train and test on the DeepWeeds dataset. An input image fixed shape resizer of 640 × 640 dimensions was used with the SGD optimizer. The batch size equal to 4 was found to be a reasonable value. With all the described settings, the model took around 14 h. The loss plot to understand the training performance of the model is presented in Figure 5A. In almost 60K iterations, the model settled to its final loss value with a small fluctuation between 0.55 and 0.75%. None of the classes achieved a satisfactory AP, and the siam weed class achieved the highest AP of 54.55% among all other classes. Three classes including lantana, prickly acacia, and negative achieved the lowest AP of 9.09, 9.09, and 0.11% respectively. Most of the testing images belonging to the negative class did not detect and the remaining images resulted in parthenium and siam weed as shown in Figure 5B.

##### EfficientDet

**Figure 6:**
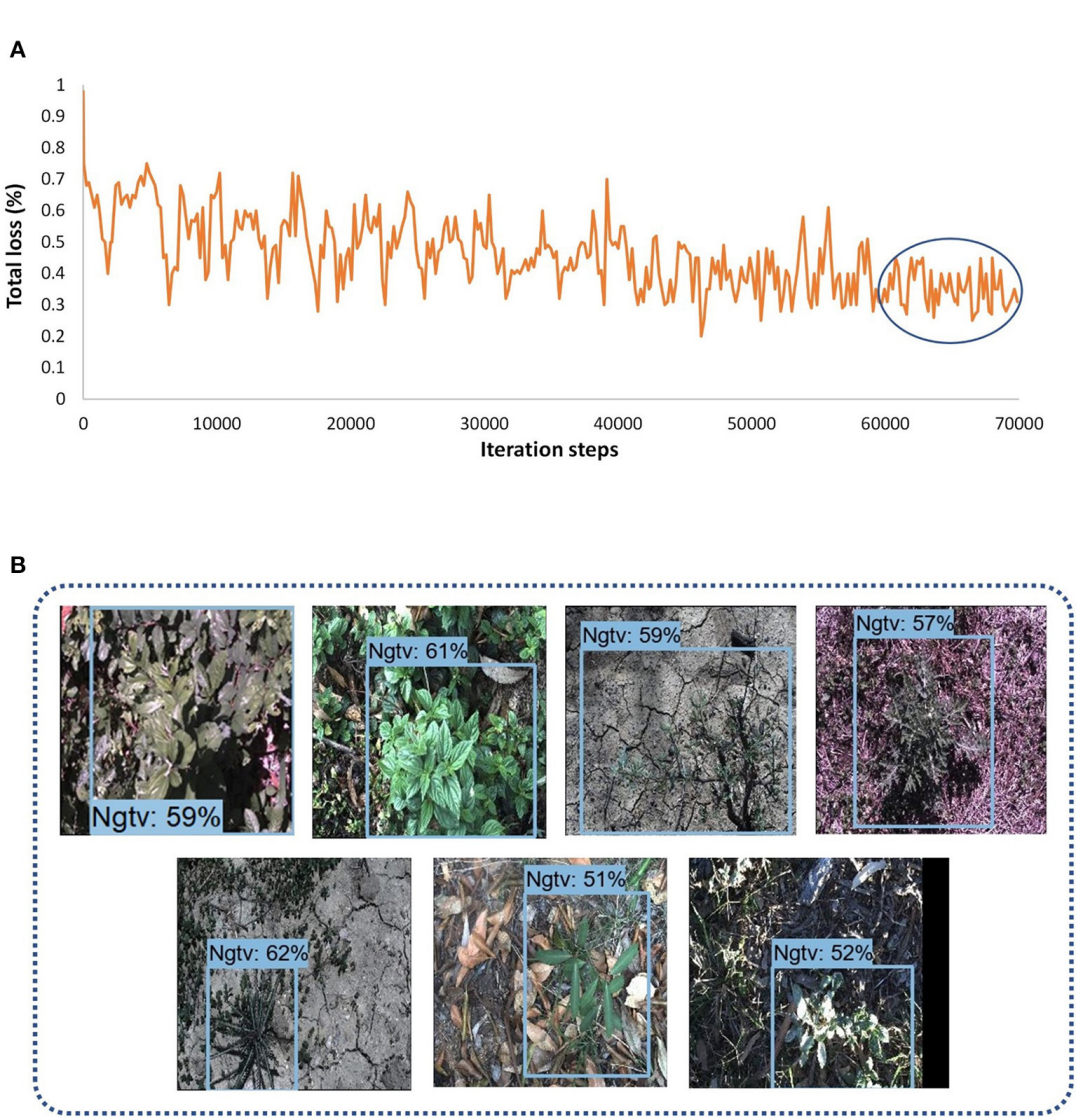
Performance of the EfficientDet model; **(A)** total loss plots; **(B)** false positives of different classes with negative class.

Another single-stage DL object detector was utilized for this study named EfficientDet. This model used the aspect ratio resizer technique with dimension 512 × 512. The other versions of the model couldn't be trained due to GPU limitations. The optimum batch size was 4 with a momentum optimizer, and this setting required 11.5 h to get convergence in the training to 70K iterations. According to the loss plot presented in Figure 6A, the model received a final loss ranging between 0.25 and 0.45%. The model also detected the siam weed with the highest AP of 63.29%. However, the prickly acacia got the lowest average precision among all seven other classes of weeds. Just like the other single-stage detectors, this DL model also gave false positives for the seven classes in terms of negative class as shown in Figure 6B. This model could also not detect any class of weeds with a higher AP.

##### CenterNet

**Figure 7:**
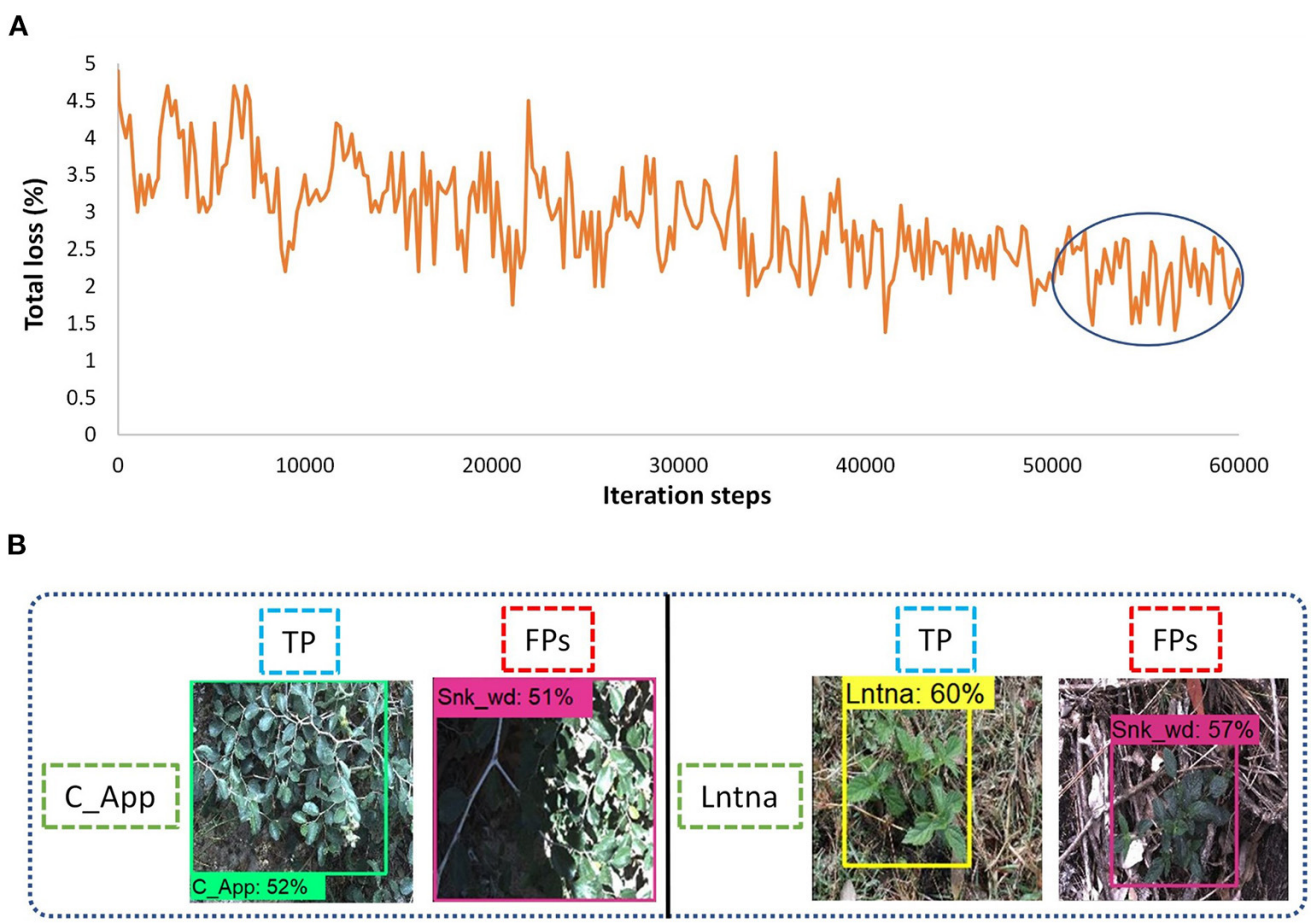
Performance of the CenterNet model: **(A)** total loss; **(B)** false-positive results for the chinee apple, and lantana classes.

The last model among single-stage DL object detectors was CenterNet which had various versions in TensorFlow API including Hourglass, ResNet-101, and MobileNet-v2. Among them, only 50 layered ResNet feature extractor was able to detect some of the weed images with 512 x 512. However, most of the images of the eight classes of weeds could not be detected well. Moreover, the SGD with momentum optimizer failed to detect the testing images with the CenterNet model. Therefore, an Adam optimizer was used to train the model, and the batch size was set to 6. The model got converged after 60k iterations and it took around 12.5 h to train the model. The final loss ranged from approximately 1.5 to 2.5%, as shown in Figure 7A. This model also achieved more than 50% average precision for the siam weed class and almost failed to detect the prickly acacia class having only 1.82% AP. However, the chinee apple and the lantana were confused with snakeweed as presented in Figure 7B.

#### Performance of Two-Stage Networks

##### RFCN

**Figure 8:**
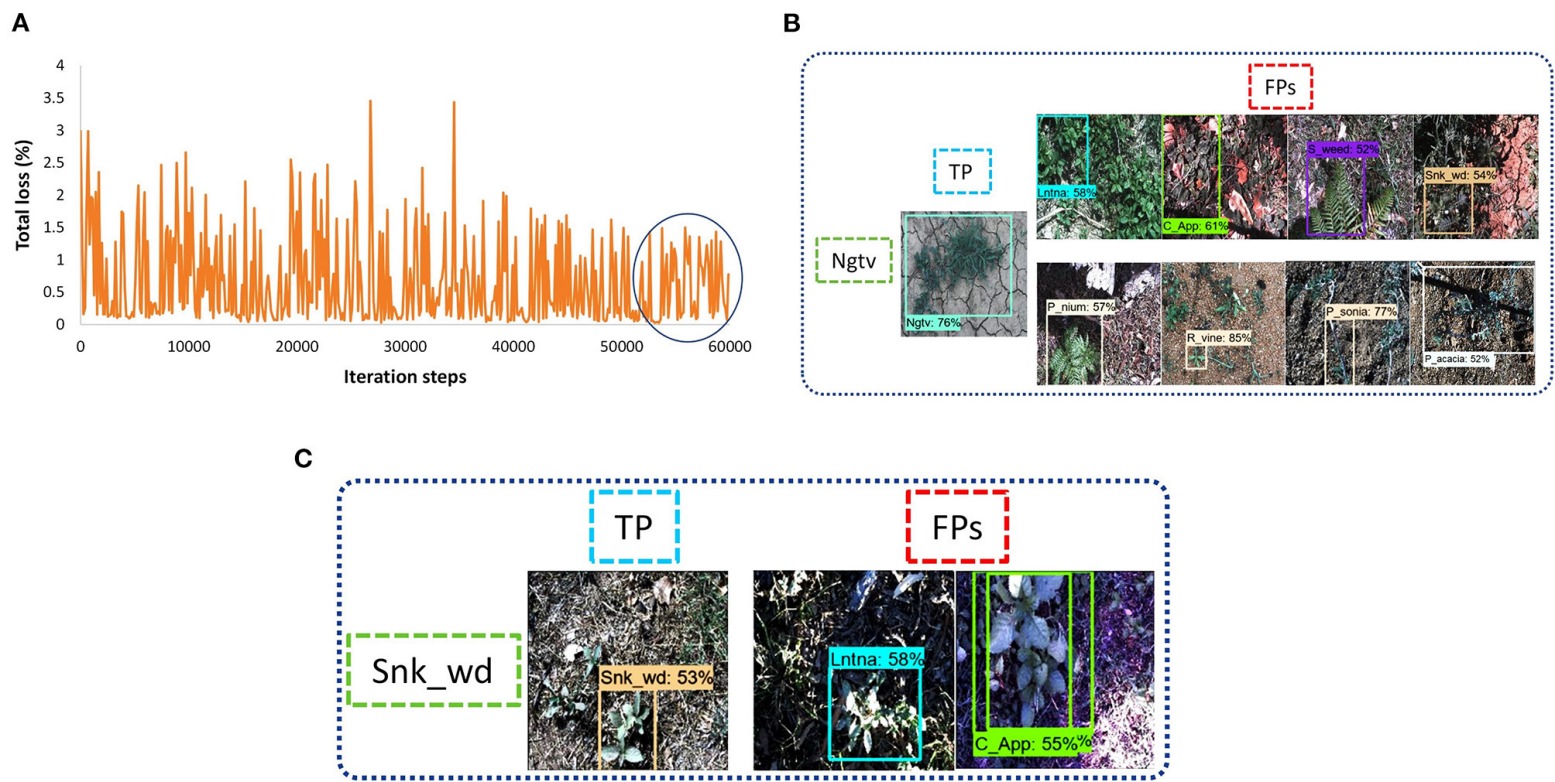
Performance of the Region-based fully convolutional network (RFCN) model: **(A)** total loss plot; **(B)** false-positive results for the negative class; **(C)** false-positive results for the snakeweed class.

This article also considers two-stage DL object detectors like RFCN and Faster RCNN. Both the RFCN and Faster RCNN models were trained to 60k iteration steps with input aspect ratio, minimum pixel size 600, and maximum 1,000. ResNet-101 was used as the backbone model and SGD with momentum optimizer was used to train the RFCN model with batch size 2. It took 10 h to get the convergence in 60K iterations. The total loss was reduced to almost 1.5% as shown in Figure 8A. The model was successful to detect three classes of weeds (lantana, parkinsonia, and rubber vine). However, classes such as negative and snakeweed were confused with the other classes, as shown in Figures 8B,C. Overall, five classes of weeds achieved an average precision of <50%.

##### Faster RCNN

**Figure 9:**
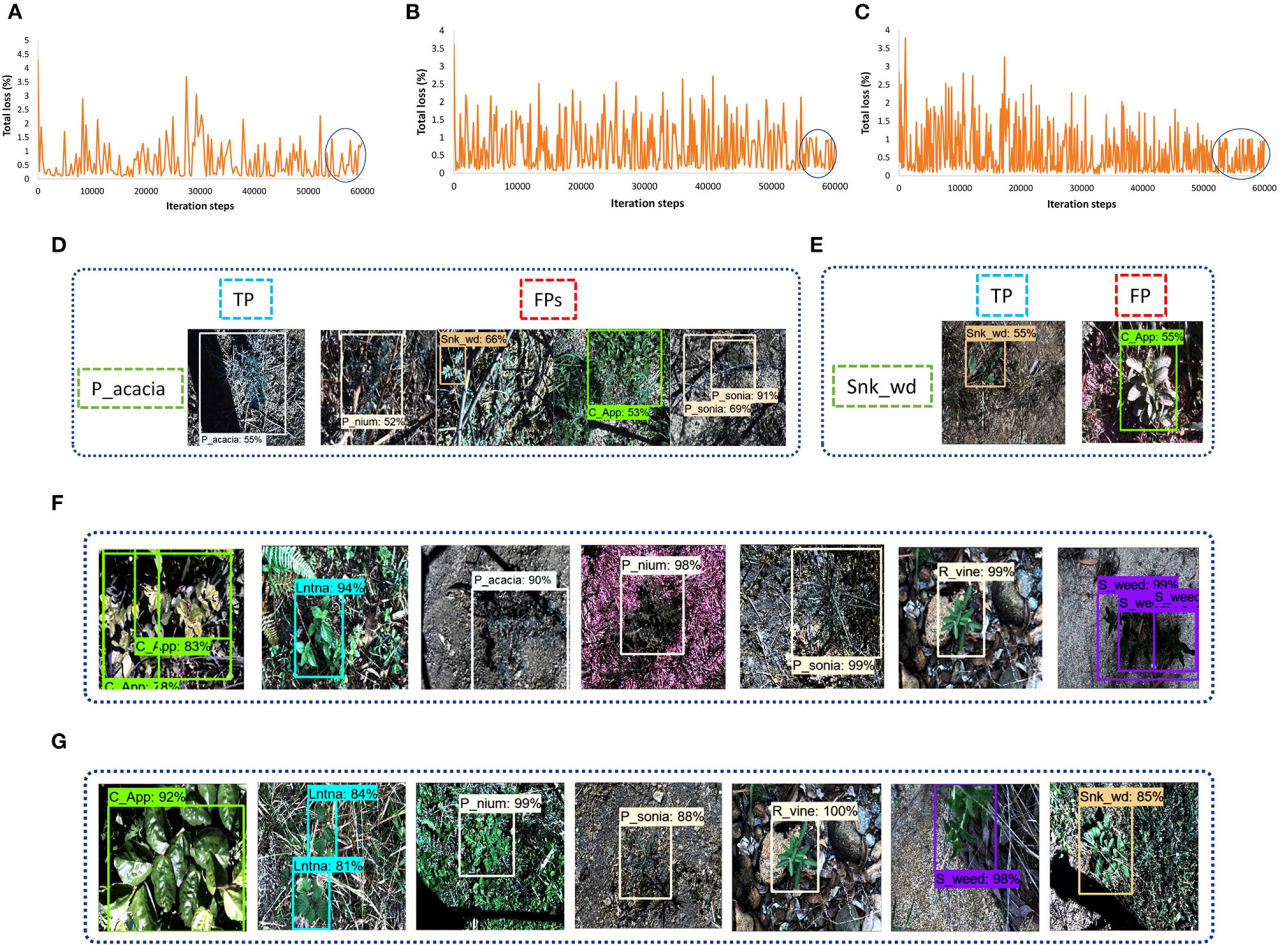
Faster Region-base fully convolutional neural network (RCNN) performance with various versions of DL models: **(A)** total loss plot for Inception-v2: **(B)** total loss plot for ResNet-50; **(C)** total loss plot for ResNet-101; **(D)** false positives of prickly acacia with Inception-v2; **(E)** false positives of snake weed classes with Inception-v2; **(F)** true positives with ResNet-50; **(G)** true positives with ResNet-101.

At last, the Faster RCNN model was trained with several feature extractors. The backbone models that included Inception ResNet, ResNet, and Inception were available along with their trained weights on the COCO dataset in TensorFlow object detection API 1. Three models, including Inception-v2, ResNet-50, and ResNet-101, were able to train with the available graphics memory. The total training loss with the Inception-v2 model was settled at almost 1.5% (as shown in Figure 9A). However, models like ResNet-50 and ResNet-101 got their convergence having fluctuation between 0 and 1%, as shown in Figures 9B,C, respectively. Furthermore, it can also be observed that the Faster RCNN model with the ResNet-101 model converged earlier than the ResNet-50 model. All versions of the Faster RCNN model had an input with the aspect ratio image resizing technique having 600 minimum and 1,000 maximum pixel dimensions, and the batch size was set to 2. Furthermore, the momentum was used to optimize the weights in this step of the proposed study; the models were trained up to 60K iteration steps. Among these three backbone models, the Inception model trained in the shortest time of around 8.5 h. However, ResNet-50 and ResNet-101 required 9 and 10 h, respectively.

For concluding the detection results, the Inception model was successful to detect five classes of weed. However, the prickly acacia and snakeweed obtained a low average precision. A few examples of both classes with their false-negative results are shown in Figures 9D,E. The ResNet-50 model detected seven classes of weeds with high AP (more than 90%) as shown in Figure 9F. The negative classes also achieved an acceptable percentage of AP as shown in Table 3. However, the snakeweed class suffered from lower precision, as it was confused with the chinee apple, lantana, and siam weed classes.

Faster RCNN ResNet-101 was found to be the most suitable DL architecture for this study due to its highest mAP compared to all other DL architectures. This model succeeded to detect four classes of weed with more than 90% average precision; three classes achieved more than 80% AP. This model achieved more than 50% AP for negative; the prickly acacia achieved the lowest AP of almost 70% among all the other classes of weeds. Few samples of images detected by the Faster RCNN ResNet-101 model are presented in Figure 9G. From the results, it can be understood that the model was successful in extracting the unique features of several classes of weeds, but could not correctly extract the features of classes such as negative and prickly acacia. Therefore, the overall performance of this model was optimized in the second step of the proposed research. It further improved the mAP along with an enhancement in the average precision of individual classes.

### Step-2: Optimization of the Faster RCNN ResNet-101 Model

The Faster RCNN model trained with the ResNet-101 feature extractor achieved the highest mAP. Therefore, the rest of the steps of the proposed work were applied to this DL architecture and the effects of image resizing techniques, weight initializers, batch normalization, and DL optimization algorithms were studied.

#### Effects of Image Resizing Techniques

This research evaluated the effects of two image resizing techniques, including fixed shape and aspect ratio, along with four image interpolation methods such as bilinear, bicubic, area, and nearest neighbor. First, the default settings of the Faster RCNN model were considered and tested using the model with the hyperparameters described in an earlier section. Later, the best image resizing method was found considering bilinear interpolation as the default method. It led to the application of three other interpolation methods. An overall mAP along with class-wise precision of each weed class was evaluated. Furthermore, various types of training losses are also presented to show the dependence of image resizers/interpolators on the performance of the DL model. The most suitable technique was selected for the next phase of the research. In this regard, the following observations are taken from this stage of the proposed methodology.

**Figure 10:**
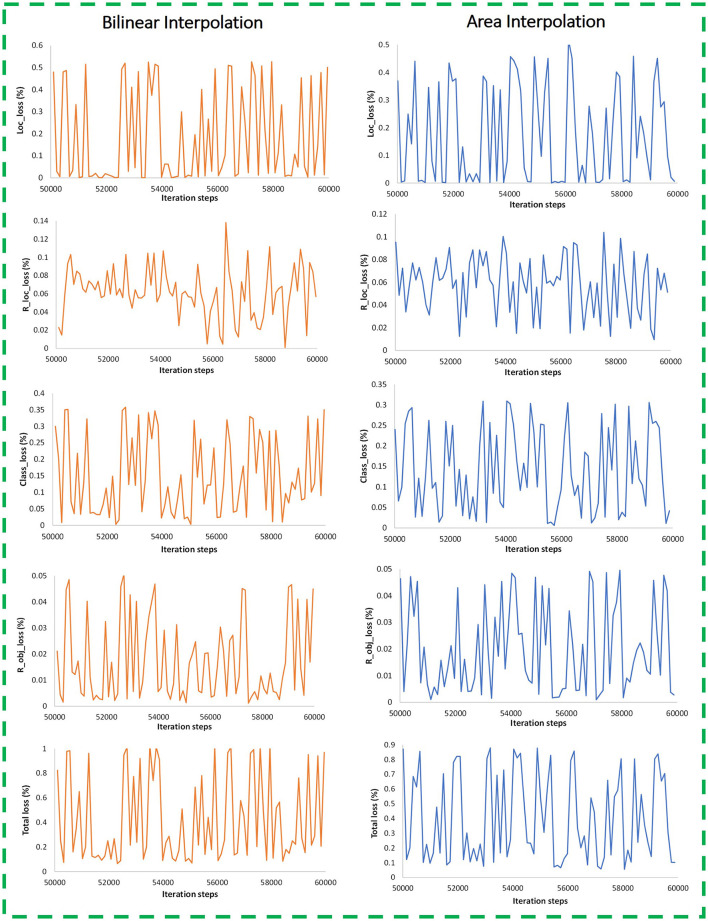
Training plots for bilinear and area interpolation methods with aspect ratio image resizer (iteration steps from 50K and onwards are shown, when the model got training convergence).

- In the Faster RCNN paper (Ren et al., 2015), the aspect ratio image resizer was selected as the default technique with minimum and maximum pixel dimensions equal to 600 and 1,000, respectively, and considered bilinear interpolation as the default method.
- The mAP of the default resizer was found to be 87.64%, as shown in Table 3.
- Although, the Faster RCNN model achieved good detection results. However, there was room to further enhance the performance of the neural network with other resizing methods.
- Later, the fixed-shape resizer method was applied. This method was first tested with bilinear interpolation, which provided a comparatively lower mAP. Furthermore, three interpolation methods like bicubic, area, and nearest neighbor with fixed image resizer attained lower mean average precision compared to the default technique, which was the aspect ratio with bilinear interpolation.
- Therefore, the aspect ratio resizer was selected as the best image resizing method for training the Faster RCNN ResNet-101 model. Here, the effectiveness of the aspect ratio resizing technique has also been validated through the experiments presented in this article, as it was also applied in the original Faster RCNN paper (Ren et al., 2015).
- The bilinear interpolation takes the closest 2 x 2 neighborhood of known pixel values and calculates the weighted average of 4 pixels to get the resultant interpolated value (Malik et al., 2017). Therefore, the pixels of weed images were interpolated to get sharper images to be provided as an input to the Faster RCNN model.
- Subsequently, three interpolation methods were also applied to the aspect ratio resizer. The bicubic method could not contribute to the improvement of mAP or detection results.
- The nearest neighbor's performance was almost similar to the bilinear method. However, the 'area' interpolation method provided significantly better training performance than the Faster RCNN model. It resulted in a higher mAP of 91.55% as presented in Table 4.
- Moreover, the average precision of the individual five classes was improved by a significant margin; these classes include lantana, prickly acacia, parkinsonia, snakeweed, and negative.
- The area interpolation method reduces noise from the images. The final images fed into the network contributed to better feature extraction of the weed classes.
- It was also noticed that the input provided to the Faster RCNN ResNet-101 model with aspect ratio resizer with area interpolation method achieved an improvement in both training and testing performance. This has been shown graphically by various losses that constitute the total training loss. The losses like RPN (Region Proposal Network) and final classifier losses are presented by RPN objectness loss (R_obj_loss), RPN localization loss (R_loc_loss), classification loss (Class_loss), and localization loss (Loc_loss) as shown in Figure 10. It is important to consider these losses in the analysis because this research is dedicated to performing the weed detection task, which contains both classification and localization operations.
- From Figure 10, it can be concluded that the losses related to the localization and classification tasks were reduced when the area interpolation method was applied. For example, the localization loss (Loc_loss) was settled with a small fluctuation between 0 and 0.52% for the bilinear interpolation method, which got reduced to 0–0.45%. Similarly, the classification loss (Class_loss) was 0–0.35% and reduced to 0–0.3%. Furthermore, RPN losses were improved with the area interpolation method.
- Since both the localization and classification losses were reduced in the region proposal and classifier stages of the network, therefore, total losses were also reduced from 0–1% to 0–0.87%. Hence, a small reduction in the model's losses produced a considerable effect on weed detection results.

**Table 4:** Summary of results and conclusions from each step of the proposed methodology.

**Experiment/step of the analysis**	**DL models**	**Training details**	**Model assessment on training and testing datasets**			**Model performance analysis**	**Link to training code**
		**(Image resizers/interpolators/** **initializers/optimizers)**	**Total loss (%)**	**Training time (h)**	**mAP (%)**		
Training with default settings	Yolo-v4	FS (608 x 608)	2.83	12	79.68	Few of the weed classes were successfully identified	GitHub
	SSD Inception-v2	FS (300 x 300)	4–6	11	48.12	None of the weed classes was succeeded in achieving an AP of more than 90%	
	SSD MobileNet-v2	FS (300 x 300)	3–6	5	34.99	Fastest model convergence, but unsatisfactory testing outcomes	
	SSD ResNet-50 (RetinaNet)	FS (640 x 640)	0.55–0.75	14	21.69	Achieved the lowest mAP among all the DL models	
	EfficientDet EfficientNet	AR (min: 512, max: 512)	0.25–0.45	11.5	36.59	Eight classes attained AP of <50%	
	CenterNet ResNet-50	AR (min: 512, max: 512)	1.5–2.5	12.5	27.36	None of the classes achieved a satisfactory AP	
	RFCN ResNet-101	AR (min: 600, max: 1,000)	1.50	10	55.06	The model was successful to detect three classes of weeds	
	Faster RCNN Inception-v2	AR (min: 600, max: 1,000)	1.50	8.5	73.59	The model was successful to detect five classes of weed	
	Faster RCNN ResNet-50	AR (min: 600, max: 1,000)	0–1	9	87.23	Seven classes of weeds with high AP (more than 90%)	
	Faster RCNN ResNet-101	AR (min: 600, max: 1,000)	0–1	10	87.64	The most suitable DL architecture for this study due to its highest mean average precision compared to all other DL architectures.	
Effects of image resizers/ interpolators	Faster RCNN ResNet-101	AR with bicubic	0–1.4	10	81.33	Could not contribute to provide better detection results	GitHub
		AR with area	0–0.87	10	91.55	Found as the best interpolator	
		AR with NN	0–0.98	10	86.93	Almost similar performance to the bilinear method	
		FS with bilinear	0–0.92	9.5	85.09	Provided a comparatively lower mAP	
		FS with bicubic	0–1.2	9.5	82.38	Attained a low mAP just like with AR	
		FS with area	0–1.5	9.5	85.68	Area interpolator did not work with fixed shape resizer	
		FS with NN	0–1.4	9.5	82.64	Attained low AP of the weed classes	
Effects of initializers and batch normalization		Tr (std: 0.01); SV (sf: 1.0, nd: true, mode: Fan_avg); RN (std: 0.01)	0–0.87	10	91.55	Very small values of std should not be taken close to zero; the normal distribution with an average of input and output units in the weight tensor should be considered	GitHub
		BN (decay: 0.99, eps: 0.01)	0–0.82	8.5	93.37	An improvement of 1.82% was obtained with BN with a fast training convergence	
Effects of optimizers		SGD with momentum	0–0.87	8.5	93.37	The default optimizer attained a high AP except for the negative clas	GitHub
		Adam	0–0.94	7.75	91.56	Faster convergence with adaptive algorithm	
		RMSProp	0–0.86	7.75	93.44	The best-obtained DL optimizer, slightly improved the mAP without BN	

*FS, Fixed-shape resizer; AR, Aspect ratio resizer; NN, Nearest neighbor; Tr, Truncated normal initializer; std, standard deviation; SV, Scaling variance initializer; sf, scaling factor; nd, normal distribution; RN, random normal initializer; BN, Batch normalization; eps, epsilon*.

In summary, an improvement in various losses along with AP (of individual classes) and mAP indicates that the area interpolation method with the aspect ratio resizer can also be applied to the Faster RCNN model for other relevant datasets. An improvement in detection output proves that the scientific community should focus on further advances in image resizing/interpolation techniques using CNN (Islam et al., 2018).

#### Weights Optimization

##### Initialization Techniques

The previous studies have been performed several agricultural tasks by using various advancements in DL including training techniques, augmentation methods, and modifying particular types of hidden convolutional layers of neural networks. In contrast, this research studied the effects of weight optimization methods on the performance of DL models.

An optimized version of the Faster RCNN model is presented by analyzing the effects of weight initializers, batch normalization (BN), and DL optimizers. Firstly, the initialization techniques were studied since they are important for a neural network to prevent vanishing gradients, which is essential to get convergence of the models (Narkhede et al., 2021). It is vital to initialize the weights with neither too large nor too small standard deviation, as both conditions fail the network to learn the features properly. Three weight initializers were studied, including truncated normal, scaling variance, and random normal initializers, according to the names presented in the TensorFlow object detection API. The optimum selection of the weight initializer parameters for the Faster RCNN model and its effects are discussed as follows.

- The truncated normal initializer is the most recommended weight initializing technique for a convolutional neural network due to the use of the ReLU activation function in almost all the networks. The reason is its vanishing gradient solving capability. This initializer is very useful for eliminating dead neurons.
- The truncated normal initializer was used to avoid any value beyond twice the standard deviation. Different values of the standard deviation of the truncated initializer affected the overall model's performance. Initially, the Faster RCNN model was trained with unit standard deviation, but it was an unsuitable value to converge the training. It can be concluded that the higher the value of standard deviation and the closer to 1, the more the training time and the lower mAP would be obtained.
- Therefore, this initializer was used with a standard deviation equal to 0.01 and a zero mean value (Ren et al., 2015). When selecting a lower standard deviation value, it should not be taken very close to zero because the mAP obtained with a standard deviation equal to 0.001 was almost 8% less than the mAP with the selected SD (0.01).
- Then, the random normal initializer was also tested that attained a mAP of 89.07%.
- Later, a scaling variance initializer with a fully convolutional layer (FC) was used. It also contains a few tunable parameters including scaling factor, normal distribution, and three modes of operation depending on input and output units in the weight tensor.
- With the combination of scaling factor 2 without normal distribution and considering only input units in the weight tensor (Fan_In), a considerably good performance in terms of losses and a mAP of 85.47% was observed.
- Furthermore, the scaling factor 1.0 considering the normal distribution of the Fan_Avg mode (which contains an average of the number of input and output units in the weight tensor) was found to be the most appropriate setting to get the best detection results in terms of mAP. Table 4 presents the mAP (91.55%) having parameter values of the weight initializer described in this section with the aspect ratio resizer by the area interpolation method.

##### Batch Normalization

The next step was to study the effects of batch normalization (BN) on the performance of the Faster RCNN ResNet-101 model. Training/testing profiles were evaluated in the absence and presence of BN. The following observations were made:

- First, the model was trained with default values of decay and epsilon of 0.99 and 0.001, respectively. The training performance of the Faster RCNN model was improved in terms of a total loss of 0.85%. The iterations were reduced to only 47K steps from 50K iterations. It shows the fast convergence of the DL architecture with the application of BN (Santurkar et al., 2018).
- However, the mAP was almost equal as obtained in the previous step of weight initialization with 91.34%.
- Next, the decay and epsilon were tuned, and it was found that a higher value of epsilon improved the overall training/testing performance of the model. For instance, at an epsilon value of 0.01 and default decay value, the mAP was improved with a margin of 1.82% as compared to the former stage of the weight initialization. The total training loss was also reduced to almost 0.82%.
- AP of individual classes was also improved including chinee apple, prickly acacia, parkinsonia, and siam weed with a difference of 22.85, 13.13, 8.99, and 9.39%, respectively. On the other hand, two classes such as lantana and negative (non-weed) attained a lower AP with BN.
- Otherwise, a smaller epsilon (0.0001) degraded the performance of the Faster RCNN model with only 76.83% of mAP. Similarly, the smaller decay could not contribute to improving the mAP and attained 88.46% with 0.5 decay value.

##### Deep Learning Optimizers

**Figure 11:**
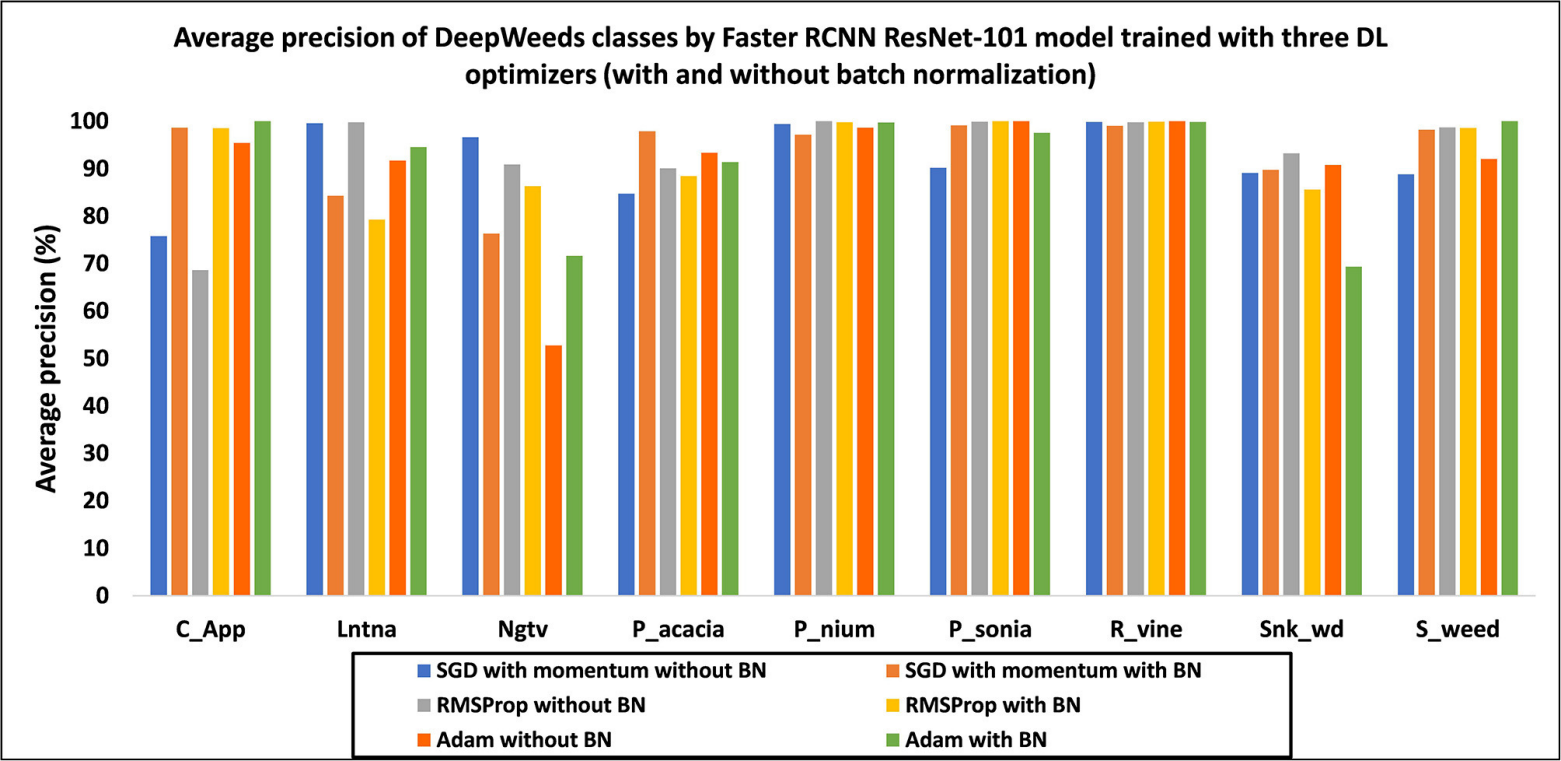
Average precision of each class by the Faster RCNN ResNet-101 model trained with three DL optimizers in the absence and presence of batch normalization.

The final step of the study presented in this article is comprised of the optimization of weights by three DL optimizers. Hyperparameters were selected using the random search method (Bergstra and Bengio, 2012) as presented in Table 2. The SGD with momentum was the default optimizer for training the Faster RCNN ResNet-101 model. Subsequently, Adam and RMSProp were used to optimize the weights of the model. A class-wise performance of the DL optimizers is presented in Figure 11. The effects of all three optimizers are discussed below.

- First, the DL optimizers were analyzed with and without BN. In the presence of BN, the SGD with momentum and Adam optimizers improved the performance with 1.82 and 1.05% in mAP, respectively, compared to the results obtained without BN. However, RMSProp got a reduction of 0.5% in mAP with BN.
- The RMSProp attained the highest mAP of 93.44% in the absence of BN. However, the SGD optimizer attained a comparable mAP of 93.37% with BN. Due to a slight difference of mAP by RMSProp (without BN), it was selected as the best DL optimizer. Moreover, RMSProp attained a much higher AP of classes including lantana and negative (non-weed), as shown in Figure 11.
- In terms of the number of training steps, the Faster RCNN model required a lesser number of steps with BN. For example, the SGD optimizer required around 47K steps to obtain the model convergence. However, adaptive optimizers including Adam and RMSProp required around 44K steps and showed a faster training convergence (Zhou et al., 2020).
- Few classes attained the same/high average precision with the three DL optimizers, such as parthenium and rubber vine. It suggests that these classes of weeds should not be addressed in future studies.
- It is also noticed that all classes achieved an AP of more than 90% except for chinee apple, when the model was trained with RMSProp. Therefore, it can be concluded that the features of this particular class of weed were not extracted through RMSProp.
- The effectiveness of the fine-tuned adaptive method like RMSProp has been shown from these experiments. Furthermore, the significance of the random search method (Bergstra and Bengio, 2012) has also been evidenced for not only the learning rate but also for the other hyperparameters, including decay factor, momentum, and epsilon (presented in Table 2).
- The stratified k-fold cross-validation technique was used to further confirm the final mAP of 93.44%. In this regard, the dataset images were redistributed in five folds (fold1-fold5). The initial distribution of the dataset was considered the first fold (fold1). The optimized Faster RCNN ResNet-101 model was retrained with the rest of the four-folds. A small difference in mAP was observed from 0.14 to 0.46%, and attained 93.30, 93.84, 93.71, and 92.98% using fold2, fold3, fold4, and fold5, respectively.

To conclude, an overall summary of all the experiments performed for this research is presented in Table 4, including the model assessment on training/testing datasets and relevant comments indicating the significance of each step taken.

## Conclusions and Future Recommendations

This article presents a deep learning-based approach consisting of five steps for the detection of weeds. First, an open-source dataset called DeepWeeds was selected due to its dynamic nature, which considered various practical aspects of an agricultural field. Next, the performance of various single-stage and two-stage neural networks was evaluated. After an in-depth analysis of the DL architectures, Faster RCNN trained with the ResNet-101 feature extractor model achieved the highest mAP of 87.64%. Later, several attempts were made to improve the class-wise average precision of the best-obtained DL model. Formerly, the effects of image resizing techniques and image interpolation methods were studied. The aspect ratio resizer with the area interpolation method attained the highest mAP of 91.55% which was 3.91% better than the default settings. Furthermore, the training performance of the Faster RCNN model was also enhanced in terms of various classification and localization losses. Next, weight-optimization techniques were thoroughly studied. In this regard, the effects of weight initializers, including truncated normal, scaling variance, and random normal, were evaluated. The performance of the model was also analyzed with batch normalization and an enhancement of 1.82% in mAP was observed. Finally, the adaptive DL optimizers including Adam and RMSProp were used to retrain the Faster RCNN model. The optimal selection of hyperparameters of the RMSProp optimizer slightly improved the mAP by 93.44%. Hence, an improvement of 5.8% mAP in the best-obtained DL architecture was achieved as compared to the default settings and it proved the effectiveness of the weed detection pipeline presented in this article.

The methodology presented in this research would be a considerable step toward precision agriculture due to a significant improvement in a complex agricultural task such as weed identification. Furthermore, this research also provides various future directions to further enhance the agricultural field of research by DL:

- The chinee apple class attained the lowest average precision with the optimized settings of the Faster RCNN model. Therefore, future research could include the modification of the Faster RCNN model to extract the unique feature of the chinee apple and maintain the average precision of other weed classes. For example, the Faster RCNN ResNet-101 contains a region proposal network and a classification model (ResNet-101). The ResNet-101 has several hidden layers to extract the distinct features of the objects. Therefore, an in-depth analysis of various hyperparameters of ResNet-101 could be performed including the number of hidden layers, filter size, number of strides, and using the latest advancement in activation functions.
- This research could be useful for other agricultural applications, including detection of plant diseases, classification of agricultural land cover, recognition of fruits, etc. After analyzing the performance of the single-stage and two-stage DL object detectors, the proposed DL-based study can be treated as an intermediate step before proposing any modification in the DL architecture.
- The weights obtained by the final optimized Faster RCNN model can be reused as transfer learning to other weed-related datasets.
- This research was dedicated to improving the final mAP of the Faster RCNN ResNet-101 model. Future research could also attempt to analyze/reduce the computation/training time and real-time detection of weeds.
- Other advanced DL optimizers can be used for upcoming studies such as Ranger optimizer.

## Data Availability Statement

The annotation files of the DeepWeeds dataset, configuration files for all models, inference graph of the final model, and dataset for five-fold cross-validation method are made publicly available in a GitHub repository https://github.com/kmarif/DL-Weed-Identification.

## Author Contributions

MHS and KMA designed the research. MHS proposed the methodology, performed the experiments, wrote the original draft, and prepared the revision. KKV curated the data and contributed to getting the results. KMA reviewed and edited the manuscript. KMA and JP obtained funding and supervised this research. All authors contributed to the article and approved the submitted version.

## Funding

This research was funded by the Ministry of Business, Innovation, and Employment (MBIE), New Zealand, Science for Technological Innovation (SfTI) National Science Challenge.

## Conflict of Interest

The authors declare that the research was conducted in the absence of any commercial or financial relationships that could be construed as a potential conflict of interest.

## Publisher's Note

All claims expressed in this article are solely those of the authors and do not necessarily represent those of their affiliated organizations, or those of the publisher, the editors and the reviewers. Any product that may be evaluated in this article, or claim that may be made by its manufacturer, is not guaranteed or endorsed by the publisher.
